# A restoration ecology perspective on the treatment of inflammatory bowel disease

**DOI:** 10.1093/emph/eoz031

**Published:** 2019-12-10

**Authors:** Matthew R Orr, Kathryn M Kocurek, Yong J Bakos, Ryder C McDowell

**Affiliations:** 1 Department of Biology, OSU-Cascades, 1500 SW Chandler Avenue, Bend, OR 97702, USA; 2 Fall Creek Internal Medicine, 2160 NE Williamson Court, Bend, OR 97701, USA; 3 Department of Computer Science, OSU-Cascades, 1500 SW Chandler Avenue, Bend, OR 97702, USA

**Keywords:** diet, dysbiosis, ecological restoration, ecosystem, gut, microbiome

## Abstract

The human gut can be considered an ecosystem comprised of a community of microbes and nonliving components such as food metabolites and food additives. Chronic diseases are increasingly associated with disruption of this ecosystem. The science of restoration ecology was developed to restore degraded ecosystems, but its principles have not been applied widely to gut medicine, including the treatment of inflammatory bowel disease (IBD). One principle of ecological restoration is that ‘passive’ restoration, which involves removing an ecosystem disturbance, should occur before attempting additional ‘active’ interventions. We discuss evidence that poor diet is principle source of disturbance in IBD, and therefore requires better attention in its research and clinical care. Another restoration principle is that higher biodiversity may improve ecosystem behavior, but this idea has not been tested for its possible importance in donor stool during fecal microbiota transplants.

**Lay summary:** In patients with chronic disease the gut microbiome behaves like a disturbed ecosystem. Principles borrowed from the science of restoration ecology identify a need to better understand the influence of diet on treatment of inflammatory bowel disease and the importance of donor diversity in fecal microbiota transplants.

## COMMENTARY

Despite some differences with natural ecosystems [1], the gut can be viewed as an ecosystem comprised of a microbe community plus nonliving components such as mucus, food metabolites and food additives [2]. Health benefits of gut microbes have inspired therapeutic manipulation of the microbiome using probiotics and fecal microbiota transplants (FMTs; Box 1) [3, 4]. The science of restoration ecology manipulates the health of natural ecosystems, but there exists little communication between restoration ecology and medicine, even though shared principles may illuminate both disciplines [2]. Inflammatory bowel disease (IBD) presents a complex problem in medicine requiring a multifactorial approach to improve patient health. Here we discuss how principles of restoration ecology generate multiple testable, but generally under tested, hypotheses that address the role of diet and gut diversity in the treatment of IBD.

A basic principle of ecological restoration is that an ecosystem cannot be repaired until the underlying disturbance causing degradation has been removed. Removal of disturbance is known as ‘passive restoration’, and can be as simple as fencing cattle away from a stream where they have trampled the bank, denuded vegetation, and caused the stream to act erosively against itself [2]. If passive restoration proves insufficient for complete recovery, then ‘active restoration’ is implemented by, for instance, planting woody vegetation [5]. Just as it would be difficult to revegetate a streambank still disturbed by livestock, it may prove difficult to restore beneficial gut microbes using active interventions such as probiotics and FMT if underlying sources of gut disturbance go untreated [2]. This line of reasoning elevates the importance of identifying environmental disturbances that cause IBD. What might they be?

Genetic factors explain only 19–26% of the hereditary variance of IBD [summarized in 4], which leaves ample potential for environmental influences. Levine *et al.* [6] reviewed the following lines of evidence identifying diet as a key underlying disturbance in IBD. First, epidemiological studies associate increased risk of IBD with red meat, fatty food, processed food and desserts and decreased risk with a diet high in fiber. Second, many of the same foods that are associated with IBD in human epidemiological studies also promote IBD symptoms in animal models. Third, exclusive enteral nutrition (EEN), which replaces whole foods with elemental liquid nutrients, leads to clinical remission in a high proportion of Crohn’s disease patients (40–80%), but partial enteral nutrition (PEN), which consists of enteral nutrition plus a regular diet of whole foods, generally does not [7, 8]—a difference widely attributed to continued intake of a regular diet [6–9]. Fourth, when the whole foods component of PEN consists of a Crohn’s disease exclusion diet (CDED), patients show clinical remission similar to subjects consuming 100% EEN [9]. The CDED excludes foods that are associated with microbiome alteration, increased intestinal permeability, impairment of innate immunity and degradation of the gut mucous layer and epithelial barrier, such as dairy, wheat, processed food, sauces, emulsifiers, canned food, packaged snacks, soda, juice, sweetened beverages, candy and baked sweets [6, 9]. Fifth, patients on 100% CDED exhibit similar remission to patients on 100% EEN [9]. Such findings have led to a model of IBD in which gut disturbance caused by poor diet is followed by microbiota disruption, then inflammation [10].

Evidence that diet is a fundamental disturbance in IBD, combined with the primacy of passive restoration in ecoystem repair, generates the hypothesis that active approaches to treating IBD such as FMT, probiotics, prebiotics, and pharmaceuticals will be more effective if diet is controlled [2]. This hypothesis is poorly tested. Few if any studies have examined interactions between specific diet regimens and other IBD interventions. Possibly because of this lack of study, the medical literature does not prioritize diet in treatment recommendations. Despite acknowledging evidence for a fundamental role of diet in IBD [11], the American College of Gastroenterology (ACG) clinical guideline for the management of Crohn’s disease in adults [12] recommends that diet may be considered as an adjunct to other therapies—and not that it should be prioritized in conjunction with other therapies, or better tested before other therapies are utilized. Moreover, the ACG guideline recommends dietary manipulation only in patients with low-risk, but not moderate-to-high-risk, disease [12]. It further minimizes diet by stating that its benefits are not ‘durable’ because symptoms reoccur upon resumption of an unrestricted diet—which is akin to stating that the benefits of diet in treating high blood pressure are not ‘durable’ because symptoms will recur if diet lapses. Strictly speaking, benefits of a restricted diet would lack durability only if symptoms were to recur while remaining on the diet, but long-term studies of the effects of diet on IBD, or long-term interactions between diet and other treatments, are largely nonexistent [6, 11].

**Box 1. Terms and abbreviations used in this article**

Active restoration—Interventions taken to restore a degraded ecosystem that go beyond mere removal of the disturbance(s) leading to degradation. For example, active restoration in a natural ecosystem may involve reseeding native plants; active restoration of a gut ecosystem may involve adding microbes via probiotics or FMT.
CDED—Developed for passive restoration of IBD [8].
EEN—Replaces whole foods exclusively with elemental liquid nutrients.
FMT—An active restoration measure that moves microbes from a donor’s gut to a diseased recipient.
IBD—Includes Crohn’s disease and ulcerative colitis.
Passive restoration—Interventions taken to restore an ecosystem that remove the original disturbance. For example, passive restoration in a natural ecosystem may involve fencing cattle or removing an anthropogenic dam. Passive restoration in the gut may involve improved diet.
PEN—Replaces whole foods partially with elemental liquid nutrients.

Recent literature reviews found no evidence that probiotics induce or maintain remission of IBD [3], whereas FMT holds some promise [4]. Although both reviews called for further research, neither they nor the studies they referenced acknowledged the possible limitations of implementing FMT or probiotics without first addressing underlying dietary disturbance. Another review and meta-analysis by Asto *et al.* [13] found that probiotics containing *Bifidobacterium* improve symptoms of ulcerative colitis, but the majority of studies it addressed administered probiotics in conjunction with pharmaceutical therapies (e.g. mesalazine, hydrocortisone), making it difficult to know the efficacy of probiotics alone.

Asto *et al.* [13] also found that few reliable studies of prebiotics or synbiotics (probiotics plus prebiotics) exist, and called for further work in this area. From a restoration ecology perspective, future work on prebiotics or synbiotics should account for the fact that mechanism(s) of diet-based disturbances in the etiology of IBD are incompletely understood. If diet-based disturbance is largely caused by an absence of food substrates that support healthy microbiome function, then prebiotics alone may ameliorate that disturbance and provide effective treatment of IBD. If, however, diet-based disturbances arise not only from an absence of beneficial nutrients but also from compounds that promote dysbiosis, then prebiotics, by failing to remove all disturbance, will be less effective.

Assessment of initial conditions is another principle of restoration ecology that may inform research in prebiotics and synbiotics. Restoration ecologists assess the initial conditions of a disturbed system in order to better identify the steps needed to restore it [14]. If a diseased gut contains healthy microbes, then they may primarily lack food substrates needed to perform their beneficial functions, and prebiotics alone may improve IBD. If, on the other hand, the microbe community has been severely compromised, then it may be necessary to administer probiotics in conjunction with prebiotics. To our knowledge, no study has compared the efficacy of prebiotics versus synbiotics in conjunction with variation in the quality of the gut microbe community.

Long-term studies of diet and IBD are lacking in part because of the difficulty of adhering to restrictive diets. To test for dietary effects on IBD and translate findings into clinical practice, patient eating behavior must be addressed. Many IBD patients think diet plays a role in their disease [11] and are responsive to dietary recommendations, but believe that their doctors underemphasize diet—the perception practitioners do not share [15]. Better physician–patient communication about diet would be beneficial because alignment among restoration stakeholders fosters project success [2, 16] and physician behavior influences the likelihood that patients will adhere to medical treatment [17]. To successfully enlist patients as stakeholders in their gut restoration, it may be necessary to consider psychological factors that promote unhealthy eating [18, 19]. To better support physician–patient communication, foods whose presence or absence cause IBD need to be better identified [6], with awareness that results may vary among individuals because of genetic differences or the inherent variability of gut ecosystems [2]. In the same way that stream restoration may require collaboration among experts in fisheries, botany and hydrology, studying and restoring gut ecosystems may require collaboration among physicians, dieticians and psychologists.

Ecological models generate additional testable hypotheses in the role of diet in IBD. Ecologists study relationships between ecosystem structure (i.e. the identity and diversity of species present) and their effects on how natural ecosystems function (e.g. biomass production, decomposition rates and nutrient flows). Mounting evidence suggests that specific microbe taxa in the gut influence health functions such as immunity, obesity, psychology and digestion [2, 20, 21]. Numerous different microbe species likely provide redundant support for each of these health functions [2], and therefore even individuals with a depleted microbiome may retain enough species to remain healthy. Mathematical models indicate that FMT success is compromised when depleted but seemingly healthy individuals are chosen as FMT donors [2], a finding we call the donor diversity hypothesis.

To our knowledge, the donor diversity hypothesis has not been rigorously tested. FMT recipients often are examined for stool microbiota diversity, but donor stool tends to be screened only for pathogenic risk factors [22]. A recent literature review written to promote more uniform methodology and reporting of FMT did not mention donor diversity [22]. We are aware of only two studies that measured donor stool diversity in FMT. One found that fecal samples pooled from multiple donors were more diverse than samples from single donors [23]; however, only pooled donor samples were used to treat patients, and therefore their efficacy compared with single-donor samples is unknown. Another small experiment involving 13 patients tested the donor diversity hypothesis using retrospective evidence. Donors who provided transplants to responders had higher microbiota species richness than donors who provided to nonresponders [24]. However, responders also appeared to have had higher baseline species richness than nonresponders, which obfuscates any effect of donor diversity.

We developed an interactive online version of the mathematical model underlying the donor diversity hypothesis that allows for manipulation of parameters such as FMT donor diversity and the probability that a microbe species establishes in an FMT recipient [25]. The interactive model reveals that large reductions in FMT success caused by using a depleted donor can be drastically improved under some circumstances with small increases in the probability that microbe species transferred during FMT establish in the recipient [25]. It is likely that a healthy diet in the recipient would promote microbe establishment given the relationship between diet and microbe diversity [2], and thus a therapeutic recipient diet could mitigate reductions in FMT success caused by poor donor diversity. Studies testing the donor diversity hypothesis should control for recipient diet to better understand its interaction with donor diversity and to minimize confounding influences on FMT success.

Higher diversity does not correlate with better health in some body sites, such as the female reproductive tract [26], and therefore the ideas raised here do not apply universally. With respect to the gut, however, insights gained from ecological restoration into treatment of IBD can (i) motivate studies that test whether passive restoration of gut health via diet improves patient outcomes from FMT, probiotics and pharmaceuticals; (ii) support research to identify the dietary factors that contribute to IBD and their possible variation among individuals; (iii) promote alignment of physicians and patients as partnering stakeholders in gut health; and (iv) justify randomized controlled tests of the donor diversity hypothesis. Scientists studying both natural and gut ecosystems have claimed that ecology is harder than rocket science [2]. Principles applied to the successful restoration of natural ecosystems merit attention for their possible contribution to the understanding and treatment of IBD.

**Conflict of interest:** None declared.
